# Evaluation of potential reference genes in real-time RT-PCR studies of Atlantic salmon

**DOI:** 10.1186/1471-2199-6-21

**Published:** 2005-11-17

**Authors:** Pål A Olsvik, Kai K Lie, Ann-Elise O Jordal, Tom O Nilsen, Ivar Hordvik

**Affiliations:** 1 National Institute of Nutrition and Seafood Research, Nordnesboder 2, N-5005 Bergen, Norway; 2 Department of Biology, University of Bergen, Thormøhlensgate 55, N-5020 Bergen, Norway

## Abstract

**Background:**

Salmonid fishes are among the most widely studied model fish species but reports on systematic evaluation of reference genes in qRT-PCR studies is lacking.

**Results:**

The stability of six potential reference genes was examined in eight tissues of Atlantic salmon (*Salmo salar*), to determine the most suitable genes to be used in quantitative real-time RT-PCR analyses. The relative transcription levels of genes encoding 18S rRNA, S20 ribosomal protein, β-actin, glyceraldehyde-3P-dehydrogenase (GAPDH), and two paralog genes encoding elongation factor 1A (EF1A_A _and EF1A_B_) were quantified in gills, liver, head kidney, spleen, thymus, brain, muscle, and posterior intestine in six untreated adult fish, in addition to a group of individuals that went through smoltification. Based on calculations performed with the *geNorm *VBA applet, which determines the most stable genes from a set of tested genes in a given cDNA sample, the ranking of the examined genes in adult Atlantic salmon was EF1A_B_>EF1A_A_>β-actin>18S rRNA>S20>GAPDH. When the same calculations were done on a total of 24 individuals from four stages in the smoltification process (presmolt, smolt, smoltified seawater and desmoltified freshwater), the gene ranking was EF1A_B_>EF1A_A_>S20>β-actin>18S rRNA>GAPDH.

**Conclusion:**

Overall, this work suggests that the EF1A_A _and EF1A_B _genes can be useful as reference genes in qRT-PCR examination of gene expression in the Atlantic salmon.

## Background

In real-time RT-PCR, the expression levels of the target genes of interest are estimated on the basis of endogenous controls. Various housekeeping genes, ribosomal RNA (rRNA) and total RNA are most commonly used as references in gene expression analysis today. The purpose of these controls is to remove or reduce differences due to sampling, i.e. differences in RNA quantity and quality. The ideal endogenous control should be expressed at a constant level among different tissues of an organism, at all stages of development and should be unaffected by the experimental treatment. It should also be expressed at roughly the same level as the RNA under study [1]. However, data normalization in real-time RT-PCR remains a real problem, especially for absolute quantification [1]. Numerous studies have revealed that no single universal gene has a constant expression level under all developmental or experimental situations. The best choice of reference gene to use as an endogenous control varies, depending on the tissues of interest in the experiment. A large number of genes have for this reason been selected for normalization of mRNA expression data [2,3]. If the selected reference gene fluctuates randomly between samples, small differences in expression between the genes of interest will be missed. Gene expression coefficient of variation (CV) between different groups of individuals should ideally be as low as possible [4]. In general, the stability of several potential reference genes should be tested in every examined tissue or cell, and under different experimental design [5,6]. An increasing number of papers are discussing the selection of reference genes in real-time RT-PCR analyses [3,7].

Two of the most commonly used reference genes are those encoding glyceraldehyde-3P-dehydrogenase (GAPDH) and β-actin. Recently, the use of these two genes as endogenous controls has been scrutinized, and several studies have documented that the GAPDH and β-actin genes should be used with caution as controls [2,8,9]. GAPDH in mammals is known to play a role in a broad range of cellular mechanisms (for review see Sirover [10]), including being a key enzyme in glycolysis. Overall, GAPDH mRNA levels might be regulated under a large number of physiological states, and its use as a reference is inappropriate for most experimental conditions. Actin is a major component of the protein scaffold that supports the cell and determines its shape, and is the most abundant intracellular protein in eukaryotic cells. Even though commonly used as a reference, the application of the β-actin gene has recently been characterized as a historical carryover from northern blots and conventional RT-PCR (for a general discussion on the use of 'classic' reference genes like GAPDH and β-actin, see Huggett et al. [7]). Eukaryotic elongation factor 1A (eEF1A, formerly elongation factor 1 alpha) plays an important role in translation by catalyzing GTP-dependent binding of aminoacyl-tRNA to the acceptor site of the ribosome. However, the protein is involved in a broad diversity of functions and constitutes 1–3% of the total cytoplasmic protein content of the cell. In human, cDNAs of two actively transcribed isoforms have been cloned (eEF1A-1 and eEF1A-2) (for review see Thornton et al. [11]). Two paralog EF1A genes (A and B) have recently been applied as references in real-time qRT-PCR of Atlantic salmon [12]. It is plausible to assume that the presence of these highly similar genes is a result of a tetraploidization event that occurred in a salmonid ancestor in the comparatively recent past [13,14].

Previously, the 18S rRNA gene was considered to be an ideal internal control in qRT-PCR analysis (Ambion [15]). Ribosomal RNA constitutes up to 80–90% of total cellular RNA, and several studies have shown that rRNA varies less under conditions that affect the expression of mRNAs (discussed in Bustin & Nolan [16]). However, questions have been raised against the use of ribosomal RNA genes as references. Vandesompele et al. [5] have stressed the fact that there sometimes might be imbalances in rRNA and mRNA fractions between different samples, making genes encoding ribosomal RNAs unsuitable as references. To meet these challenges of accurate interpretation of real-time qRT-PCR data, the authors suggested that an index of the most stable housekeeping genes should be used for normalization, and developed the *geNorm *VBA applet for Microsoft Excel in this regard [5]. A similar software tool, the *BestKeeper*, has been developed by Pfaffl et al. [6]. These tools can be used to find the most stable reference genes under different experimental conditions. We used the *geNorm *software which determines the individual stability of a gene within a pool of genes [5]. The stability is calculated according to the similarity of their expression profile by a pair-wise comparison, using their geometric mean as a normalizing factor. The gene with the highest *M*, i.e. the least stable gene, is then suggested excluded in a stepwise fashion until the most stable genes are determined, and an index suggested, based on the best genes. *geNorm *has been used to select the most stable reference genes in several recent studies (e.g. [4,17,18]).

The aim of this work was to evaluate the usefulness of six potential reference genes in the Atlantic salmon. Salmonid fish are among the most widely studied model fish species in general, and extensive basic information on many different aspects of their biology has been collected [19]. Large-scale DNA-sequencing projects on salmon have been initiated in several laboratories ; ; ; . In this work we selected the two 'classic' reference genes encoding GAPDH and β-actin, two genes encoding 18S rRNA and S20 ribosomal protein and two paralog genes encoding elongation factor 1A (EF1A_A _and EF1A_B_). To evaluate their usefulness as reference genes, RNA from eight tissues of six adult salmon were subjected to real time PCR. The relative transcription levels of the genes were also estimated in four phases of young salmon going through smoltification, in order to check their stabilities under physiological stressful conditions.

## Results and discussion

### Ranking of six potential reference genes in Atlantic salmon

The ranking of the six examined genes analyzed by *geNorm *is shown in Table 3. In six tissues (muscle, liver, gills, head kidney, spleen and thymus), the EF1A_B _gene emerged as the most stable, whereas the EF1A_A _gene was ranked number one in brain and the β-actin gene was ranked number one in intestine. The 18S rRNA and S20 genes were ranked among the worse genes in all tissues. Not surprisingly, the GAPDH gene was ranked worse in five tissues (liver, head kidney, spleen, brain and thymus), confirming the general skepticism against the use of this gene as reference [7,16,20]. Combined, the total ranking reads EF1A_B_>EF1A_A_>β-actin>18S rRNA>S20>GAPDH. We did not analyze our data with the *Bestkeeper *software. Analyzing reference genes in virus infected cells, Radonic et al. [4] concluded that the *Bestkeeper *tool gave results that slightly deviated from, but nevertheless corresponded to, those obtained using *geNorm*.

**Figure 1:**
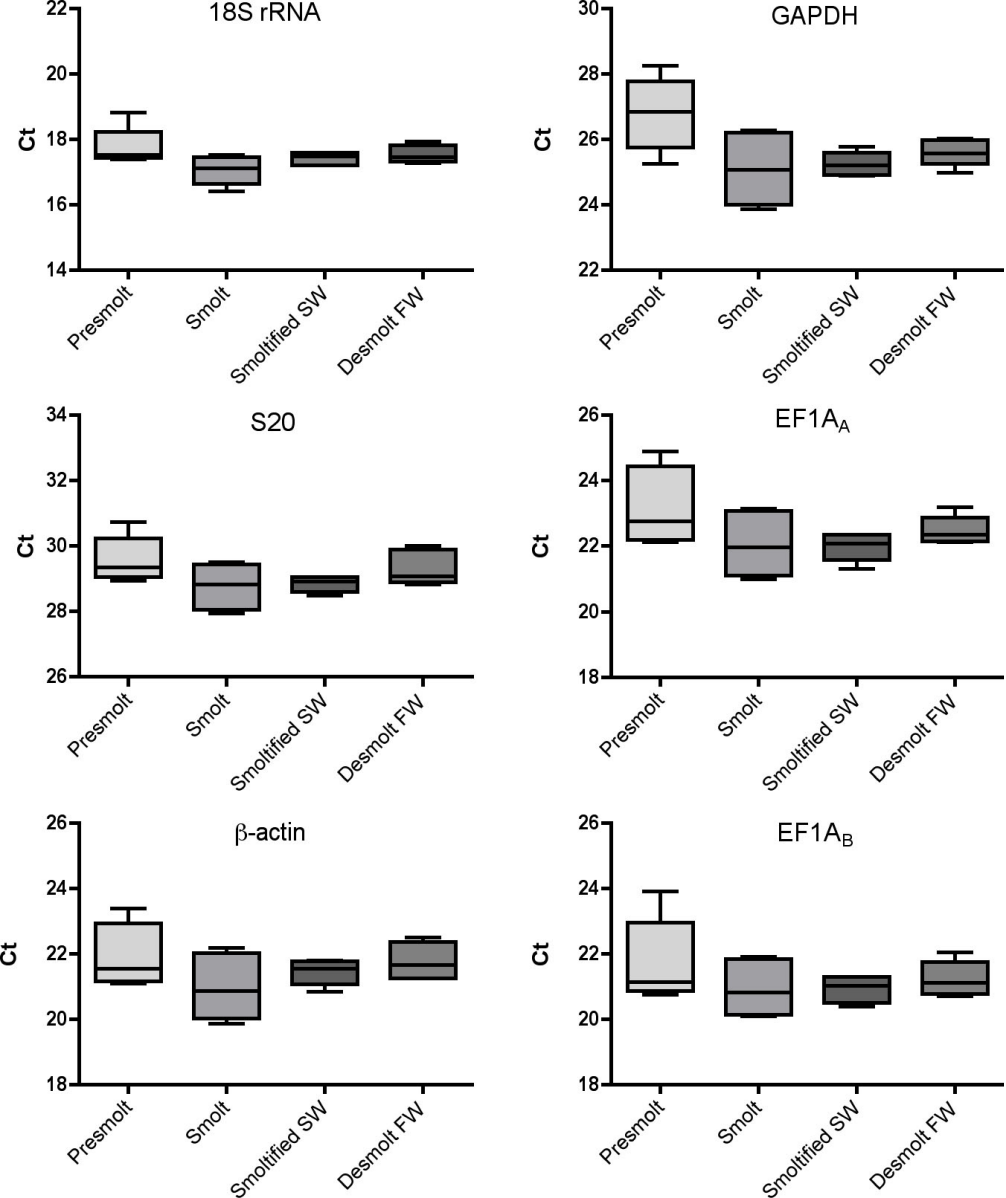
qRT-PCR analysis of six genes in gills of six Atlantic salmon going through smoltification; presmolt (before smoltification), smolt (during smoltification), smoltified (finished smoltified in seawater) and desmolt (desmoltification in freshwater). Numbers indicate raw Ct values.

**Figure 2:**
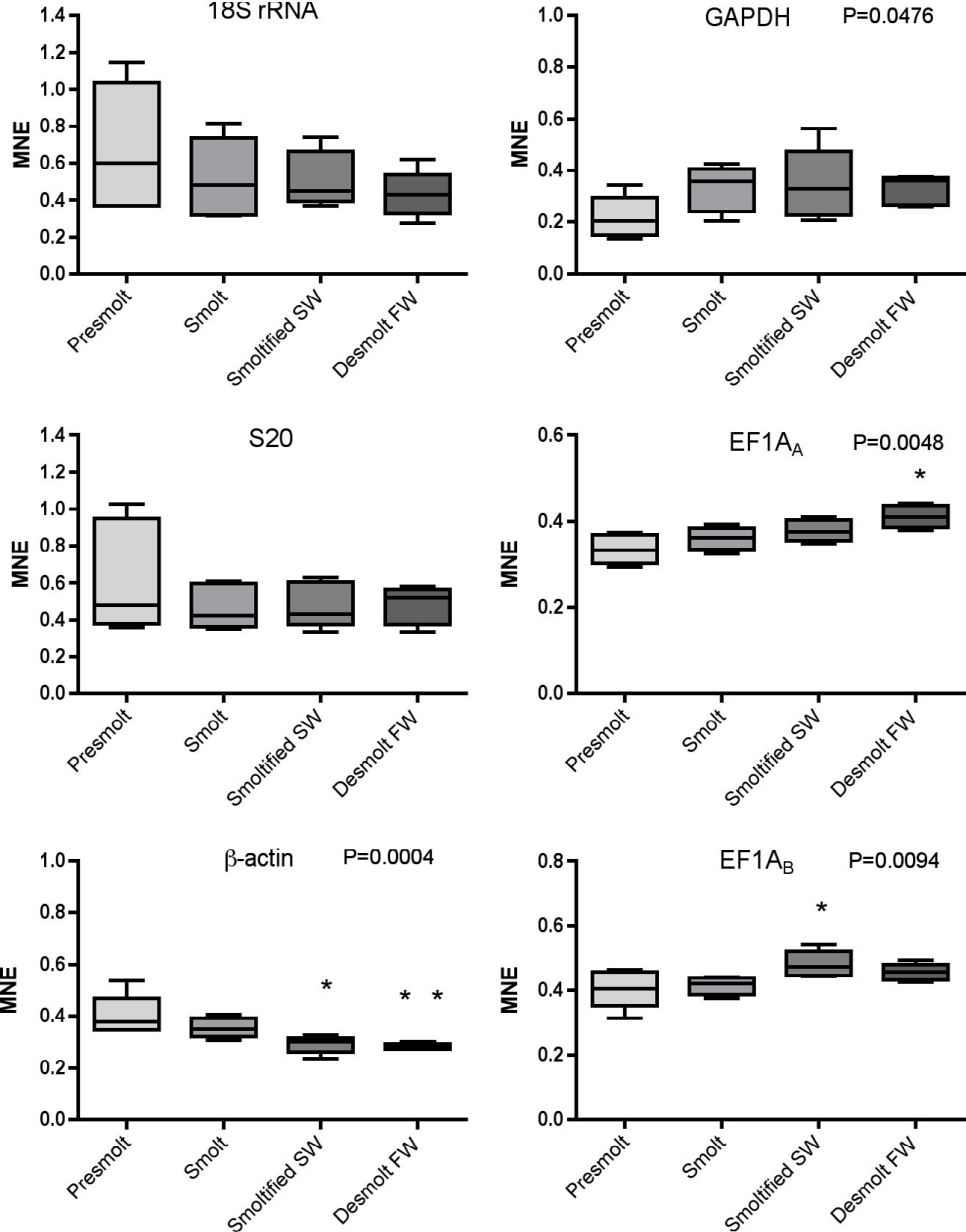
qRT-PCR analysis of six genes in gills of six smoltifying Atlantic salmon. The same data as in Figure 1, but now normalized against an index of the three best genes (β-actin, EF1A_A _and EF1A_B_) calculated with the *geNorm *software. The four groups were analyzed with Kruskal-Wallis test, and if significant, the overall p-value is given in the graphs. For β-actin, there were significant differences between the presmolt and the smoltified group (p < 0.05), the presmolt and the desmolt groups (n<0.01) and between the smolt and desmolt groups (p < 0.05). For EF1A_A_, there was a significant difference between the presmolt and the desmolt groups (p < 0.01). For EF1A_B_, there was a significant difference between the smolt and the smoltified groups (p < 0.05). An asterisk denotes significant differences between the groups.

**Figure 3:**
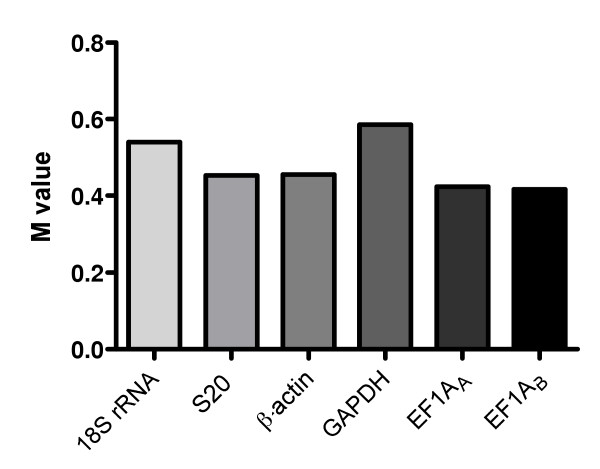
Stability of six genes in gills of Atlantic salmon during smoltification calculated with the *geNorm *software. *geNorm *calculates the gene expression stability measure M for a control gene as the average pairwise variation V for that gene with all other tested control genes.

To be able to evaluate gene stability under stressful conditions, mRNA expression of the selected genes was examined in gills of salmon going through smoltification. Prior to seawater entry, juvenile anadromous salmon undergo a parr-smolt transformation, characterized by behavioral, morphological and physiological changes, known to be challenging for the fish. Physiological alterations include increased seawater tolerance, olfactory sensitivity, metabolic rate, scope for growth and changed hemoglobin and visual pigments [21]. We selected to examine the gills during smoltification, because this tissue plays a major role in ionic and osmotic regulation during adaptation to hyperosmotic seawater. Figure 1 shows the raw Ct values of the studied genes in gills before, during and after smoltification (smoltified in seawater and desmoltified in freshwater). In Figure 2 the same data are presented, but now normalized against an index calculated by *geNorm *of the three most stable genes (β-actin, EF1A_A _and EF1A_B_). Based on the M values, *geNorm *ranks the stability of the six genes from 24 fish going through smoltification in the following order: EF1A_B_>EF1A_A_>β-actin>S20>18S rRNA>GAPDH (Figure 3). In Figure 1 it can be seen that the 18S gene had the lowest individual raw Ct variation. Most individual raw Ct variation of the studied genes is seen in the presmolt and smolt groups. A characteristic drop in expression can be seen for all genes in the smolt group, compared to the presmolt group. After transfer to seawater, the individual raw Ct variation decreased for all genes. Overall, the raw Ct data suggest that the physiological challenging smoltification process affected the expression of all six genes. When the same data were normalized against an index of the three most stable genes, β-actin, EF1A_A _and EF1A_B_, the relative expression levels were altered for all genes. Now the ribosomal 18S gene emerges as the second worse, whereas the two paralog EF1A genes became the most stable. This might have to do with the fact that geNorm will top-rank co-expressed genes [22], a weakness that has to be considered when evaluating paralog genes likely to be co-regulated. Even though the eEF1A-2 gene has been identified as an important oncogene and has been shown to be differently expressed in human tissues [11], Hamalainen et al. [23] found the eEF1A-1 gene to be a good reference gene in real-time RT-PCR examinations. A similar finding was reported by Frost and Nilsen [24] in salmon louse, where they showed that the eEF1A and S20 genes were valid candidate references, whereas the 18S rRNA and GAPDH genes were unsuitable. The current findings based on *geNorm *evaluation question the recommended application of ribosomal genes as references (as suggested for example by Ambion (see reference [15]), and are in line with earlier warnings against the use of rRNA genes as references [5,6]. To avoid the normalization of the genes for β-actin, EF1A_A _and EF1A_B _against an index partly based on their own expression, the S20 gene was included in the index instead, and the mean normalized expression for these three genes calculated with the new index. The patterns of expression, however, were approximately the same for the three genes as seen in Figure 2, suggesting that the gene-stability measure M can be used to find the most appropriate reference genes.

### PCR poisoning

We see a correlation between the A260/230 absorbance on the NanoDrop and the PCR efficiency (data not shown). We tend to get PCR efficiencies that are too high in some samples with low A260/230 ratios. When the samples are treated with DNase solution, the A260/230 ratio usually drops. After DNase treatment, the A260/280 ratio increased from 1.8 to 2.1 (n = 45 samples). At the same time, the A260/230 ratio dropped from 2.4 to 2.1. The DNase treatment therefore adds substances to the RNA solution that increases the absorbance at 230 nm more than it decrease the 260 nm absorbance. The added substance (salt or some other component) may inhibit the RT reaction or the PCR reaction, sometimes called PCR poisoning. We have seen that the A260/230 ratios are quite low in samples that give inadequate PCR efficiency slopes, especially with RNA from head kidney, thymus and intestine tissues, in which the gradient of the standard curve is less than -3.3 (Table 2). The reason one obtain better amplification rate efficiencies with the more diluted samples is because the inhibitor has been diluted below its effective level. The obvious way around this problem is to dilute the amount of cDNA put into the PCR reaction. Alternatively, cleanup columns can be used to purify and concentrate the RNA.

Transcription levels of the examined genes and the coefficient of variance (CV) in different tissues varied considerably. mRNA levels in tissues are regulated by numerous endogenous and exogenous stimuli [16]. Transcription rates in metabolic active tissues might be up-regulated compared to those of less active tissues, whereas inter-tissue variation in degradation rates of mRNAs, for example, might affect mRNA stability [25]. The results revealed that muscle had the lowest CVs of the studied genes, compared to higher CVs in more active tissues like thymus, head kidney and spleen. In thymus, intestine, head kidney, gills, brain, liver and spleen, the 18S and S20 genes had the lowest CVs, based on raw Ct values. In all tissues, except intestine, the GAPDH gene had the highest CV. Except for thymus, the two elongation factor genes had relatively similar expression in all eight examined tissues. Their expression are most likely co-expressed in the examined tissues, and therefore favored in *geNorm *calculations [22]. The results also demonstrated that assays optimized for one tissue of an organism do not necessarily work equally well in other tissues. Of the tissues studied in this work, intestine, head kidney and spleen were the most troublesome.

## Conclusion

Our data, based on *geNorm *calculations, suggest that the Atlantic salmon EF1A genes that have been tested in the present study may be good candidate reference genes. The GAPDH gene seems unsuitable as a reference in quantitative real-time RT-PCR. With regard to the 18S rRNA gene, this must be applied with caution. Tools like the *geNorm *applet for Microsoft Excel can be useful to help select the most stable genes in various experiments.

## Methods

### Fish handling and experimental design

Tissues from 15 individuals were collected (852 ± 702 g, ranging from 254 to 1898 g). These individuals were not separated based on sex, size or sampling time, but treated as one heterogeneous group to examine the width of mRNA expression of the studied genes in eight different organs. This group of fish was handled and fed according to normal aquacultural management, and none of these individuals were exposed to any particular treatment. To examine if physiological stress may alter the gene expression in the gills, a total of 24 individuals were collected before (termed presmolt, 18.3 ± 0.9 g), during (termed smolt, 28.7 ± 3.7 g) and after smoltification. After smoltification, one group was kept and desmoltified in freshwater (termed desmolt FW, 30.0 ± 3.8 g), while the other group was transferred to seawater (termed smoltified SW, 30.2 ± 4.3 g) (n = 6 in each group). The Atlantic salmon examined during smoltification were from the anadromous population "Vosso" of the river Vosso in Southwestern Norway (see Nilsen et al. [27] for details on how these fish were treated). All fish were treated and euthanized according to Norwegian national legislation for laboratory animals.

### Tissue sampling

Samples from eight organs, i.e. gills, liver, brain, head kidney, spleen, thymus, white muscle and posterior intestine, were dissected out and immediately frozen in cryo tubes in liquid N and stored at -80°C before RNA extraction. The RNA extracted from three spleen and four head kidney tissue samples were of low quality, and we had to redo the sampling from four individuals, These tissue samples were stored on RNA later (Ambion) at -20°C before further processing.

### RNA extraction

RNA was isolated with phenol-chloroform extraction as described by Chomczynski and Sacchi [28], and stored in 100 μl RNase-free MilliQ H_2_O. Total RNA was extracted using Trizol reagent (Invitrogen, Life Technologies), according to the manufacturer's instructions. Genomic DNA was eliminated from the samples by DNase treatment according to the manufacturer's description (Ambion). The RNA was then stored at -80°C before further processing. The quality of the RNA was assessed with the NanoDrop^® ^ND-1000 UV-Vis Spectrophotometer (NanoDrop Technologies, Wilmington, DE, USA) and the Agilent 2100 Bioanalyzer (Agilent Technologies, Palo Alto, CA, USA). A 260/280 nm absorbance ratio of 1.8 – 2.0 indicates a pure RNA sample. The RNA 6000 Nano LabChip^® ^kit (Agilent Technologies, Palo Alto, CA, USA) was used to evaluate the integrity of the RNA. We used the RNeasy MinElute Cleanup kit from Qiagen to purify our most troublesome samples. With this kit the A260/230 ratio increased on average by 5 % (n = 10).

### Design of PCR primers and TaqMan probes

The PCR primer and TaqMan MGB probe sequences used for quantification of the genes encoding 18S rRNA, S20 ribosomal protein, β-actin, GAPDH, EF1A_A _and EF1A_B_, are shown in Table 1. Four of these genes, 18S, β-actin, EF1A_A _and EF1A_B_, have also been used as references in real-time RT-PCR analyses of Atlantic salmon in other recent studies [12,28]. The primers amplify PCR products between 57–98 basepairs (bp) long, which is within the range of 50–150 bp as suggested by Applied Biosystems for their TaqMan assays. qPCR assays were designed using Primer Express 2.0 software (Applied Biosystems, Foster City, CA, USA) to select appropriate primer and probe sequences from known Atlantic salmon genes. The mRNA sequences encoding S20 ribosomal protein and GAPDH were obtained from GenBank accession numbers BG936672 and BU693999, respectively (exon-exon borders were not considered). The EF1A_A _assay was based on a cDNA clone that we reported to the GenBank previously (AF321836), whereas the EF1A_B _assay was based on the EST BG933853. An alignment with zebrafish indicated the exon-exon borders [29]. The chosen primers were subsequently used to confirm that the salmon genes contained an intron between the same sites as deduced from the alignment with zebrafish. The PCR products containing the introns were cloned into TOPO vector (Invitrogen) and sequenced (sequences can be provided upon request). PCR primers for β-actin were based on Atlantic salmon BG933897 and designed to span exon-exon borders of this gene, as deduced from corresponding genes in human and zebrafish (NW633959). For 18S rRNA the PCR primers and probe were designed from the Atlantic salmon sequence AJ427629, and placed in a conserved region of the gene based on comparison with the human gene. RNA samples were subjected to DNase treatment to avoid genomic DNA contamination. Amplified PCR products of all actual cDNAs were sequenced to ensure that the correct mRNA sequences were quantified. The fragments were sequenced with BigDye version 3.1 fluorescent chemistry (Applied Biosystems) and run on an ABI PRISM^® ^377 DNA apparatus at the University of Bergen Sequencing Facility.

### Real-time quantitative RT-PCR

A two-step real-time RT-PCR protocol was developed to measure the mRNA levels of the studied genes in eight tissues of Atlantic salmon. The RT reactions were run in triplicate on 96-well reaction plates with the GeneAmp PCR 9700 machine (Applied Biosystems, Foster City, CA, USA) using TaqMan Reverse Transcription Reagent containing Multiscribe Reverse Transcriptase (50 U/μl). Two-fold serial dilutions of total RNA were made for efficiency calculations. Five or six serial dilutions (500 – 15,63 ng) were analyzed by qRT-PCR in separate sample wells and the resulting Cts recorded. Input total RNA concentration was 500 ng in each reaction for β-actin, GAPDH, EF1A_A _and EF1A_B_, and 0.5 ng for 18S rRNA and S20 ribosomal protein. Controls for no template (ntc) and controls for no amplification (nac) were run for each master mix, but not for every single sample. Reverse transcription was performed at 48°C for 60 min by using oligo dT primers (2.5 μM) for β-actin, GAPDH, EF1A_A _and EF1A_B _and random hexamer primers (2.5 μM) for 18S and S20 in 30 μl total volume. cDNA for evaluation of smoltification effects (24 samples) were primed entirely with random nonamer primers. In this material, total RNA were treated with RQ1 RNase-free DNase (Promega) and cDNA reversely transcribed using 500 ng total RNA and random nonamers in conjunction with the Reverse Transcription Core kit (EuroGenTech, RT-RTCK-05) following the manufacturer's instructions. Input total RNA concentration was 500 ng in each reaction. All 24 samples for each gene were run on the same plate together with six serial dilutions. The final concentration of the other chemicals in each RT reaction was: MgCl_2 _(5.5 μM), dNTP (500 μM of each), 10× TaqMan RT buffer (1×), RNase inhibitor (0.4 U/μl) and Multiscribe Reverse Transcriptase (1.67 U/μl).

2.5 μl of 10-fold diluted cDNA for β-actin, GAPDH, EF1A_A _and EF1A_B_, and 1000-fold diluted cDNA for 18S rRNA and S20 ribosomal protein from each RT reaction was transferred to a new 96-well reaction plate, and the real-time PCR run on the ABI Prism 7000 Sequence Detection System from AB. Real-time PCR was performed by using TaqMan Universal PCR Master Mix, which contains AmpliTaq Gold^® ^DNA polymerase, and gene specific primers (900 nM). Fluorescence marked TaqMan MGB probes (200 nM) were used for data collection during the log linear phase of the reaction. PCR was achieved with a 10 min activation and denaturation step at 95°C, followed by 50 cycles of 15 s at 95°C and 60 s at 60°C. Baseline and threshold for Ct calculation were set automatically with the ABI Prism 7000 SDS software version 1.1, or set manually whenever necessary.

For evaluation of the potential reference genes, raw Ct values are presented. The *geNorm *VBA applet for Microsoft Excel was used to determine the most stable genes from the set of tested genes [5]. The Ct values were transformed to quantities using standard curves, according to the *geNorm *manual. The gene expression stability (M) was calculated with the *geNorm *applet, and the genes were ranked from best to worst, based on the M value.

### Statistics

The GraphPad Prism 4.0 software (GraphPad Software, Inc.) was used for the statistical analyses in this work. Linear regression was used to determine PCR efficiency based on dilution curves. Non-parametric Kruskal-Wallis test was used to compare differences among four groups of salmon going through smoltification.

## Authors' contributions

PAO was responsible for the experiment, data analysis and drafted the manuscript. KKL conducted the real-time RT-PCR analysis, and contributed throughout the experimental process. AEOJ constructed the qPCR assays for two of the genes. TON provided the cDNA from the smoltification experiment. IH participated as a supervisor in the study design, analyses and writing.

## Figures and Tables

**Table 1:** TaqMan assays used to evaluate potential reference genes in Atlantic salmon.

Gene	Forward primer	Reverse primer	TaqMan MGB probe	Amplicon size (bp)
18S rRNA	CCCCGTAATTGGAATGAGTACACTTT	ACGCTATTGGAGCTGGAATTACC	CACCAGACTTGCCCTCC	98
S20	GCAGACCTTATCCGTGGAGCTA	TGGTGATGCGCAGAGTCTTG	CCTCAAGGTGAAGGGA	85
β-actin	CCAAAGCCAACAGGGAGAAG	AGGGACAACACTGCCTGGAT	TGACCCAGATCATGTTT	91
GAPDH	AAGTGAAGCAGGAGGGTGGAA	CAGCCTCACCCCATTTGATG	CTGATCATTGGAAACCT	96
EF1A_A_	CCCCTCCAGGACGTTTACAAA	CACACGGCCCACAGGTACA	ATCGGTGGTATTGGAAC	57
EF1A_B_	TGCCCCTCCAGGATGTCTAC	CACGGCCCACAGGTACTG	CCAATACCGCCGATTTT	59

**Table 2:** Standard curve evaluation of potential reference genes in Atlantic salmon.

	Thymus		Muscle		Intestine		Head kidney	
	Slope	R^2^	Slope	R^2^	Slope	R^2^	Slope	R^2^
18S rRNA	-3.26	0.996	-3.40	0.993	-3.23	0.992	-3.27	0.998
S20	-3.28	0.996	-3.21	0.991	-3.29	0.990	-3.07	0.993
β-actin	-2.73	0.994	-3.36	0.997	-3.11	0.997	-3.05	0.996
GAPDH	-3.38	0.997	-3.21	0.995	-3.37	0.996	-3.13	0.996
EF1A_A_	-3.06	0.996	-3.05	0.993	-3.05	0.994	-3.06	0.996
EF1A_B_	-3.11	0.998	-3.13	0.998	-3.20	0.993	-3.31	0.997

								

	Gills		Brain		Liver		Spleen	
	Slope	R^2^	Slope	R^2^	Slope	R^2^	Slope	R^2^

18S rRNA	-3.41	0.999	-3.32	0.992	-3.42	0.998	-3.20	0.995
S20	-3.29	0.990	-3.22	0.991	-3.39	0.991	-3.28	0.995
β-actin	-3.10	0.999	-3.21	0.997	-3.23	0.990	-3.44	0.994
GAPDH	-3.57	0.997	-3.48	0.902	-3.21	0.995	-2.83	0.714
EF1A_A_	-3.38	0.998	-2.86	0.994	-3.11	0.994	-3.15	0.992
EF1A_B_	-3.32	0.991	-3.14	0.991	-3.24	0.991	-3.52	0.996

**Table 3:** Evaluation of the usefulness of six potential reference genes in eight tissues of Atlantic salmon ranked by the *geNorm *software. 1 = best, 6 = worst. Six individuals were analyzed for six genes in eight tissues.

Tissue	Muscle	Liver	Gills	Head kidney	Spleen	Brain	Intestine	Thymus	Total ranking
18S rRNA	3	5	6	2	3	5	6	4	4
S20	6	4	5	5	2	3	5	5	5
β-actin	2	3	4	4	4	4	1	2	3
GAPDH	5	6	3	6	6	6	3	6	6
EF1A_A_	4	2	2	3	5	1	3	3	2
EF1A_B_	1	1	1	1	1	2	2	1	1
